# Cancerous Degeneration of Warts

**Published:** 1852-10

**Authors:** Richard G. H. Butcher

**Affiliations:** Examiner on Anatomy and Physiology in the Royal College of Surgeons in Ireland, Surgeon to Mercer’s Hospital, &c., &c., &c.


### SELECTIONS

FROM

### FOREIGN AND HOME MEDICAL JOURNALS.

---

*On the Cancerous Degeneration of Warty Excresences, and their Treatment.* By Richard G. H. Butcher, F. R. C. S. I., Examiner on Anatomy and Physiology in the Royal College of Surgeons in Ireland, Surgeon to Mercer's Hospital, &c., &c., &c.—*Mr. President:*—On a former occasion I had the honor of bringing before the notice of this Society (the Surgical) a paper on the relationship that is found to subsist between cancer and fungus hæmatodes; and illustrated this alliance by preparations and specimens—1stly, when the diseases co-existed together; 2ndly, where the one was consecutive to, or replaced by, the other; and 3rdly, where the two manifestations of disease were tinuous in the same tumor.

These facts are borne testimony to and established by the investigations of Langstaff (Med. Chir. Trans., vol. ix.) Cruveilhier (Anat. Pathol., livr. xxiii., Explanation of Plates, 5 and 6,) and others.

The observations which I now wish to lay before the profession are in reference to the cancerous degeneration of warty excrescences—an association which I do not think has met with all the careful attention from writers to which it is entitled. Entitled on two grounds—1st, from the frequency of the one as a sequence to the other; and 2ndly, from the inveteracy of the connexion when once established.

The following cases will afford exposition of the various changes brought about, from the apparently innocent verruca to the cancerous ulcer, and this again to the contamination of the system and the springing up of encephaloid disease. To illustrate still further this subject, I shall lay before the Society numerous casts and drawings, accurate representations of the respective changes as they were effected in each individual case, and shall conclude with a few practical deductions from the premises obtained.

Case 1.—Anne Sullivan, aged fifty-two, applied to me for relief, in May, 1850, being then suffering severely from a large, painful, ulcerated tumor over the right eye. The history which she gave goes to prove that a wart about the size of a pea existed above the eyebrow ever since she was a child; that eleven months previous to her seeking my advice, it became painful and itchy; that she frequently tried to pick it away in little pieces, and often pulled long shreds out of it, the separation of which was always attended with sharp pain, lasting frequently for a lengthened period after, and usually with a smart flow of blood. About this time, too, the bulk of the swelling began rapidly to increase, with a red margin round it, and soon its appearance was altered in every respect from the original condition; the warty excrescence was cast off, and a small ulcerated surface, about the size of a shilling, lay exposed, which was elevated, hard, and circumscribed; yielding a thin yellowish discharge, and characterised by persistent pain of a pricking kind, subject at different times to various degrees of intensity. Day after day the tumor continued to enlarge, spreading its base by the accession of fresh nodules, which never rose to any greater height than half an inch above the surrounding healthy parts; the integuments thus appeared to ulcerate around, the destroyed part being supplanted with firm elevations, which, in their turn, coalesced, became convex, and in this way preserving the nodulated character of the entire surface. Thus the base extended widely in all directions —upwards on the forehead, inwards and beyond the mesial line, externally towards the temple, and down upon the cheek, and inferiorly so as to involve and depress the upper lid, and compromise vision in the right eye. The extent of ulcerated surface measured round its circumference ten inches. This amount of disease, then, was hurried into existence in the incredibly short period of eleven months. The character of the sore was peculiarly cancerous, the surface being nodulated, hard, and firm almost as cartilage, yielding a discharge thin, yellowish, and watery; profuse in quantity, and emitting the peculiar odor so pathognomonic, and readily recognized by the surgeon accustomed to meet with this form of disease. Eight months after the commencement of the disease in the forehead, a tumor began to form in the upper part of the parotidean region; it gradually came on, at first attended with most severe darting pain through the ear, up along the side of the head, and forwards towards the face, and thus averting sleep for nights, even before there was any appreciable swelling. At this time, the pain, she states, to have been most agonizing; but it gradually declined as the bulk of the tumor was augmented. The size of this secondary growth obtains in magnitude about that of a split orange, and from its osseous boundaries its outline is not regular; it is also somewhat compressed transversely about its centre, and the upper part is more of an ovoid, while the lower portion is lobulated, and spread out. This cast, taken from the patient, most faithfully represents the appearances of the primary and secondary formations, and the coloration of each has been very carefully preserved. From a careful consideration of the phenomena attendant on this tumor, the rapidity of its growth, the character of the pain, the elastic sensation elicited by the touch, the color of its surface, I concluded it was of encephaloid formation, and referred it to that class. With anxiety I watched this creature for some time, and in about five weeks after the cast was taken, the most prominent part gave way, and a fungus shot out, never attaining beyond the size of a large fig, and emitting from its centre, at intervals of a few days, repeated arterial hæmorrhages; some of them to the extent of several ounces. She struggled on in this way for two months, when she died from the debility consequent upon those frequent losses, and I regret to say I could not obtain any dissection of the body. I examined the structure of the original tumor several times with the aid of the microscope, and which most clearly proved its cancerous nature. A fine section of it showed the basis to be made up of fibrous tissue, having imbedded, as it were, in its structure numerous nucleated cells; many with nucleoli. The addition of acetic acid had no other effect than that of rendering more conspicuous the nuclei at the expense of almost the dissolution of the cell-wall. On subjecting a piece of the tumor to pressure, a juice could be expressed from it yielding an abundance of cells similar to those visible in the section, and by the addition of acetic acid were acted on with a similar result. Numerous granular bodies were also floated through the fluid.

Here is a drawing of the microscopic appearances of the primary tumor, showing the arrangement of the fibrous tissue, cells, and granular bodies, which I have adverted to. The next point to be cleared up in this case was, carefully to ascertain the nature of the secondary formation, the tumor behind the jaw, and to trace out the affinity between it and the antecedent true scirrhus by microscopic examination. After the tumor had burst and the fungus shot out, I introduced a grooved needle into its structure about an inch deep, then rotated it between the finger and thumb, and on withdrawing it the groove was loaded with the morbid product. This was not uniformly of the same consistence; some parts were harder than others. On placing a small portion of it under the microscope, every atom absolutely teemed with a profusion of nucleated cells, supported with the most delicate filamentous tissue. On examining some particles firmer than others, the cells were much the same, the only difference being in the compression of the cells; while those of the softer portions approximated more closely to a sphere. There were no caudate corpuscles present in this specimen.

Here is a drawing of the appearance of the cells, as represented under the same power as that used in the first picture.

Case 2.—Jane Murphy, aged seventy, a healthy-looking country woman, who had been mother of ten children, consulted me in January, 1849, for a small tumor situated beneath her chin in the mesial line. She mentioned that a wart had been there from childhood, but that within the last four months it had lost its form, the irregular surface becoming smooth, its size larger, and extremely painful. She had been in the habit of frequently pressing the tumor, endeavoring to allay the pain, which often induced it to bleed, and then the annoyance in a measure subsided. When I first saw this patient, the tumor was about the size of a marble, smooth and polished on the surface, with a semi-transparency over it, of stony hardness and quite moveable. Taking these features into consideration, together with the characteristic pain, always of a lancinating nature, the altered aspect of the part, and the period of life at which it was brought about, I was led to the inference of malignant degeneration being set up in this change, and urged its immediate removal. Coexisting with this suspicious tubercle, there was a warty growth, larger than a pea, a little above the chin, and to the left side. This, she said, also existed from infancy, never gave her any uneasiness, and exactly resembled the one beneath the chin, previous to the alteration above noticed. I removed the tumor beneath the chin in January, 1849, by two elliptical incisions, their long axis from above downwards cutting far wide of the diseased structure, and deeper by several lines of the bed of the tumor. The wound inflicted readily admitted of being brought together from side to side, and the edges retained so by two fine needles and the twisted suture, compresses were placed beneath the ends of each needle with a double object, to bear off any undue pressure and to act as on the principle of the quill suture in supporting the lips of the wound at their very deepest line in contact, and thus taking the strain off the needles. So effectual was the snpport and apposition afforded that union by the first intention was constrained, almost through its entire track, the lower part only suppurating. In ten days after being cut, the wound was altogether healed, and the patient went to the country to her friends. Previous to her going home, I urged the removal of the wart above the chin, but to no effect; she would not submit to have it done. During nine months after the operation, she remained free from disease, and satisfied that a cure had been effected. About the end of this time, the wart, which had been permitted to remain, began to spread and get painful. The cicatrix resulting from the former operation became tender, tumid, and ultimately gave way by an ulcerated fissure, which rapidly grew wider, yielding a profuse ichorous discharge.

The destructive action progressed for about a fortnight, when a fungus growth spread around the sulcus formed in the first instance, assuming the shape of a mushroom and the size of a crown-piece, its margin being turned over so as to rest upon the sound skin. She came up to town again for my advice, and I declined interfering by operation; the grounds of objection being chiefly founded on the presence of a deep sinus leading backwards towards the line of lymphatics, parallel and beneath the anterior margin of the sterno-mastoid muscle. Again, the root of the disease was struck so deep, and the width of the contaminating base so widely spread, that even the most expert operator could not be satisfied that the entire was removed. Palliatives were again ordered, and she returned to the country. For many months the disease very slowly increased, but the warty excrescence was very considerably augmented, its surface having ulcerated, and the same process spread its margin, until ultimately it joined the disease spreading upwards from beneath the chin, the two having coalesced and become inseparably united together. During the last four months still further changes have been added; not only has the original manifestation of the disease been progressive, but we have formed two additional tumors, situated one on either side of the neck, and in the line of the absorbents, manifestly of encephaloid nature. Their springiness and elasticity, their coloration, and above all, the microscopic examination of their contents on exploration, pointed to, and confirmed the opinion of, their being true cephaloma. In this miserable state she endured, the gravity of the symptoms having been greatly increased, pain giving rise to the most intolerable suffering, the features being haggard and pinched, and the skin of a dull ochry color, debility and emaciation having made rapid progress, and all the functions of the economy more and more becoming implicated in the deteriorating influence of the disease.

In this deplorable condition (in December, 1851), she went back to her family in the country, to await her final release from suffering, which, to all certainty, was not far distant.

Here is a cast accurately showing the condition of the parts previous to operation in January, 1849; and here is a second, graphically illustrating the changes which have been brought about, from the period of nine months after the operation, when the disease appeared in the cicatrix, with all the progressive changes up to the present time (January, 1851), an interval of fifteen months having elapsed. The painting of each has been most truthfully executed.

I have also preserved these microscopic drawings, taken of the primary and secondary tumors as they appeared. Here is one representing the appearances of the tumor that first showed itself beneath the chin. It exhibits a number of true cancer cells, scattered everywhere through a fibrous basis. Some separate cells are also seen detached.

This second drawing shows the arrangement of the encephaloid tumors which sprung up beneath the mastoid muscles. The structure seemed entirely composed of myriads of nucleated cancer-cells, and very closely resembled the secondary formation in the case of Sullivan; inasmuch as there were no caudate corpuscles in this specimen either, and the cells were held together by the finest areola tissue.

Case 3.—Ellen Fitzpatrick, aged sixty-five, consulted me in March, 1850, for a large bleeding wart, placed above and behind the right ear; it was attended for some time before with repeated hæmorrhages. She said it had been there for many years, never created any annoyance until about six weeks before seeking my advice. She referred the great change which had taken place in it to a bruise occasioned by a water pail that she had been in the habit of carrying on her shoulder. Shortly after this "the wart became very sore," and soon the pain set in, of intense character, darting up along the side of the head, down towards the angle of the jaw, and represented by the sufferer as "indescribably severe." On examining the part, a highly irritable and inflamed base surrounded the tumor, which was about the size of a shilling, uneven on its surface, and elevated about half an inch; it was hard to the touch, and bled upon the slightest pressure from an ulcerated line partly round it and through its structure.

I removed this tumor with great care, cutting far wide of the base, and as I thought most effectively. Two arteries sprung which required ligatures, and so free had been the excision that the edges of the wound would not permit of being brought together, yet it healed perfectly in three weeks by granulation, a soft yet polished cicatrix being left. For a period of eight months she continued quite well and exempt from all annoyance. After this time she began to complain of uneasiness behind the angle of the jaw on mastication; by degrees the part became tense, and then she felt a small tumor there. This at the time she believed originated from cold, and it did not alarm her, more particularly as she often relieved the urgent pain by repeated stuping. However, the swelling continued to increase so as to become perceptible, and when it attained such magnitude as to fill up the angle of the jaw, she began to suffer from the effects of paralysis of the facial division of the 7th nerve on the right side. Day after day the tumor extended itself, particularly in the direction of the site of the original warty excrescence. At this time she again sought my advice, and then the case was truly a lamentable one. A tumor, considerably larger than an orange, filled up the space between the angle of the jaw and the mastoid process, lost upwards towards the zygoma, passing downwards and encroaching on the neck, extending behind the ear, and implicating the structures attached to the occipital bone: uneven, projecting, and lobulated on its surface; fixed, irregular, and immoveable at its base. The color of the tumor was very remarkable and strikingly indicative of the condition so frequently associated with the proper circulation of the true cephaloma. Large veins traversed it in every direction, some of them lying, as it were, in grooves embedded on its surface; while again numerous vessels marked the coloration in a peculiar way, constituting what might be called a number of vascular spots, from which capillaries radiated in every direction for a short distance, and ultimately breaking up into a fine ramiform distribution.

Here is a cast taken from the patient at this time, which most accurately shows the position, form, and color of the secondary tumor, also the paralytic condition of the corresponding side of the face, from the implication of the motor portion of the 7th nerve with the morbid product.

The face is greatly distorted, and the right side very remarkable when contrasted with the other. Upon the forehead the integuments lie flat, smooth, and at rest, there being no wrinkles or motion as on the left side. A verticle furrow is placed nearly in the centre, dividing the bulging of the muscles on the left side from the uncontracted state of those on the right; and the slip of the occipito-frontalis muscle forms a remarkable prominence at the junction of the nasal bone with the frontal on the left side. The power of closing the eyelids of the right eye was lost; they remained always open. When asked to close the eye forcibly, although she made the attempt, there was not the slightest motion observed in the eyelids. When the eye was at rest, and the patient using the sound one, about half the pupil remained visible, but during sleep was completely concealed behind the upper lid. The conjunctiva of the eye was in a chronic state of inflammation, and exhibited through a lens a perfectly villous surface, permeated in every point with innumerable vessels. On close examination, the cornea looked dull, but at a little distance presented a borrowed brilliancy from the abundant flow of tears which were constantly secreted and pouring over the cheek. The lower eyelid drooped a little, and the mucous membrane lining it presented the same vascular arrangement as that covering the sclerotic coat. The right nostril lay flat, collapsed, and not distended on a deep inspiration, but rather closed together, and the nose pointed towards the left side. When she blew or attempted to whistle, the air escaped by the right angle of the mouth, the right buccinator not at all corresponding in action with the muscle of the left side, nor with that of the muscles of the chest and neck by which the air was expelled. In mastication, the food collected in the right cheek between it and the teeth, and the patient could not push it from its place without the assistance of the tongue, and frequently of the finger. The saliva constantly flowed out at this side, and when drinking, part of the fluid likewise escaped.

When the disease attained the size represented in the cast, it did not at all increase so rapidly as at first; and during the following thirteen months I had repeated opportunities of watching the course of the disease, a part of it ulcerated, a fungus shot out, and was attended by small hæmorrhages. I regret to say in January, 1852, this creature took typhus fever from an individual in the same lodging house, and died on the tenth day. I could not obtain permission for an examination of the parts.

It may be said, the cases of cancerous degeneration which I have brought forward all occurred in patients of advanced life. In most of the instances which have fallen to my lot for observation, it was so; but I have also seen the change brought about in early age, which the following cases will testify.

Case 4.—Maria Williams, aged nineteen, a particularly handsome girl, of dark complexion, consulted me in February, 1849, for what appeared a very irritable wart, and situated on the forepart of the neck. She mentioned it had been there as long as she could remember, but that latterly it had increased and became very painful, which she attributed to the pressure of her dress. The tumor when I saw her was the size of a filbert, hard and irregular on the surface, which at the highest point was elevated about a quarter of an inch above the surrounding healthy skin. It was quite moveable, placed about the centre of the depression, situated above the sternum, and three quarters of an inch from its upper margin.

The patient suffered great uneasiness in her mind from the rapidity of its increase, and the "dread of cancer," as her mother had died of that disease, and great depression and annoyance from the constant pain present in it.

Mr. Tagert, whom I consulted in the case, agreed with me that it was better to remove the part,—a proposition in which the patient most readily acquiesced. I did so by two incisions, one on either side, and wide of its base, meeting above and below, and then, by a few touches of the knife lifted the tumor in its perfect integrity from the subjacent cellular tissue. The lips of the wound were brought together with two fine needles and the twisted suture. Union by the first intention was nearly accomplished on the fourth day, and in less than a fortnight the part was healed altogether. During the three years which have elapsed, I have several times seen this young woman, and up to the present date there has been no return of the disease, either in the cicatrix or elsewhere.

I regret to say I have mislaid the microscopic drawing of the tumor cut out in this case, which I made most carefully; and more particularly so as bearing on a question about which I think a good deal of uncertainty still exists. From my notes, however, the following are the particulars. The specimen yielded epithelial scales, in various conditions and stages; some compressed together, forming laminæ, whilst deeper ones assumed a somewhat square form, some of them a caudate shape, whilst around the base there were other cells which I at once pronounced to be cancer-cells. When separated and broken up they did not at all seem disposed to run together, they were nucleated, some with nucleoli, and which, on the addition of acetic acid, were rendered more distinct, and the cell-wall was nearly dissolved, while the other cells resisted its action with impunity. I am quite sure I was not led astray here by an appearance that frequently takes place—namely, the enlargement of the epithelial cells from endosmosis.

Mr. Wardrop records a very remarkable instance of this cancerous degeneration of a wart occurring in a subject much younger than in the case which I have just related. "I had an opportunity (writes this eminent pathologist) of seeing an example of a true cancerous sore in a girl about twelve years of age, and it is the only case of the kind which has come to my knowledge. It appeared on the lower part of the abdomen, and begun in the form of a black wart on the skin. The wart ulcerated, and the surrounding skin was gradually destroyed, so as to form an immense ulcer, having all the characters of a true cancerous sore, which at last destroyed the child."—(*Wardrop's Observations on Fungous Hæmatodes, p.* 189.)

Case 5.—The supervention of fungous hæmatodes, after the removal of a large wart from the inner side of the foot, is well exemplified by the following case which occurred in our hospital some time since:—Mary Murphy, aged twenty-eight, admitted into Mercer's Hospital, October, 1846, being the second time this year. In the preceding February she was received into the house for the removal of a large painful wart, fully the size of a half-crown piece, and situated on the inner side of the left foot. It occasioned her great pain, and was so irritable that even a stocking could not be worn over it, and it was deeply ulcerated round its base. At this time there was no evidence of internal disease, and the lymphatic glands of the extremities were neither indurated nor enlarged; therefore Mr. Tagert removed the part, and without difficulty, for it had no deep attachment whatever; it was quite loose, and readily floated on the surface from the slightest touch. The wound quickly healed, and in three weeks she returned to the country. Her second admission, as above dated, was nine months after this operation, when she was received with far advanced encephaloid disease in the groin of the same side. The history which she gave of the tumor in the groin is as follows: For five weeks after her return home—that is, two months from the period of the operation—she was free from all disease; that exactly at this time "a kernel" appeared in the left groin; it continued to increase for a month, and attained the size of a small apple, when it remained stationary for a short time. Up to this period there was very little uneasiness in the part. After this the tumor began again to enlarge, with a "bursting sensation" in it. During the following months her sufferings were greatly augmented, the tumor widely extending itself in all directions, irregular and nodulated on the surface, and highly sensitive. At this time, too, just before admission, the most prominent part burst, from which she lost a quantity of blood. In this state, then, she was received nine months after the operation, the tumor being larger than the clenched hand, accompanied by darting pains occasionally through it; but she refers an indescribable sensation of tension being always located in the upper half of it, and here, too, was a black spot marking the site from which the hæmorrhage had proceeded a few days before.

November 10th.—Since her admission to hospital, the increase of the tumor has been most rapid; it is now enormous, measuring ten inches and a half transversely, and seven and a half from above downwards. Its color is also greatly altered, being now of a dark purple and reddish hue all over. Its surface is irregularly lobulated, and deprived of skin, with the elevations coated over by a semi-opaque fluid, and the depressions containing unhealthy watery pus. The constitution is sympathizing acutely with this mass of local disease. The pulse is never under 120; she has at intervals during the night profuse perspirations; her countenance is haggard and of a yellowish hue; and all appetite is gone. One point in the upper part of the tumor is far darker than the rest, and from which point two ounces of venous blood trickled the evening before. There has been no return of the disease on the foot, but the cicatrix is very hard and firm.

13th.—There was hæmorrhage last night to about two ounces, but it was readily restrained by a few dossils of lint steeped in spirits of turpentine and finger pressure.

18th.—Had profuse hæmorrhage last night; she lost nearly a pint of dark blood; to-day she is greatly exhausted, and bathed in sweat; her pulse weak, yet throbbing, and 130 in the minute; the tumor is quite black and turgid from where the blood flowed last night, and all its lobulated and broken up surface seems a mass of sloughs; she does not complain of pain now.

19th.—Is much depressed to-day; at six o'clock in the evening bleeding began again, at first slowly, and was staunched by pledgets of lint dipped in muriated tincture of iron. In two hours after it broke out afresh, and was perfectly uncontrollable. At this time the bleeding was frightful, it issued out in large bursts from the pultaceous disorganized mass. When pressure was made over one point, it welled up as rapidly from under another lobe of the fungus, and so on until death threatened by hæmorrhage; she was waxy pale, with violent jactitation of the arms, profuse cold sweat over the entire body, screaming for the windows to be opened and the admission of air. In these efforts at length all motions ceased, and though there was no appearance of life, yet the blood continued to flow for a few seconds longer, when the pulse forsook the heart, and then death.

On examination of the body, a tumor as large as a small melon, of the same nature as that in the groin, filled the iliac fossa of the same side, intimately attached to the fascia, and implicating the muscles in this region. The iliac artery and vein ran through its base, and below Poupart's ligament the femoral artery and vein were surrounded by the encephaloid structure situated there. This pathological condition may account for the fact of the total uselessness of pressure over either of the trunks in arresting the fatal hæmorrhage. On slitting up the artery and vein through their entire extent as they traversed this diseased mass, I could not by the closest examination find any so, lution of their integrity. Vessels of considerable sizeboth arteries and veins, however, could be discovered through the structure, with their opened up and patulous extremities. These were very numerous, and evidently the source from which the blood issued in such quantities. The patulous condition of the arteries, as well as the veins, I ascribe to the matting of the coats of the vessels with the surrounding tissues, and thus neutralizing their contractile power. The softer parts of the tumor, on section, exactly resembled the brain in a state of decomposition.

Case 6.—The late Mr. Palmer, of this city, had a case very analogous to the one just particularized, a short time before under his care, in Mercer's Hospital. The patient was a young woman only twenty-four years of age; she had a flat painful wart on the inner side of the knee; it was there for years, but having become very irritable and ulcerated, and bleeding from the least injury, she solicited for its removal; it was taken away by the knife, and the part healed favorably. She returned to the hospital in five months after; the glands in the groin of the same side being enormously enlarged, and all the structures in the inguinal region participating in the encephaloid degeneration. This creature died before the end of the seventh month after the operation, of repeated and profuse hæmorrhages.

Now the cases which I have given are examples of only one condition of the skin preparatory to ulceration and malignancy; that is, when there exists an indurated warty tumor, and this I conceive to have a cancerous tendency, *ab initio.* The small growth may be unproductive of inconvenience for years, until irritated, as illustrated in many of the cases which I have adduced; then the characteristic pain, sharp and lancinating, never entirely deserts it; ulceration sets in, making breaches round its base, and proceeds to the detachment of the warty surface. During this time, a thin fluid exudes from underneath; hard firm granulations are thrown up from an indurated base, not rising very high, yet presenting a mammillated surface, far denser than the interior of the projecting nodules. The destructive process which I have endeavored to describe and elucidate by the foregoing cases, presents to the inquirer two very striking characteristics, and essentially belonging to it—1st, that when once the ulcerative process is set up, there is never any amelioration, ever so temporary, no attempt at cicatrization; and 2nd, the great liability of the appearance of encephaloid disease, either in the site of the original tumor or in the line of the absorbents, returning from its position. Here, then, are two marked differences as to the results between it and the condition to which the term *noli me tangere* is applied, and to the *destructive ulceration* most accurately described by Dr. Jacob. Of this latter disease, I present to the Society this highly painted cast to contrast with those I have already exhibited. It shows well the characters of the disease as recorded by that gentleman. In this instance, though nearly half the scalp was destroyed, though inroads had been made by the disease to a considerable extent on the side and posterior part of the neck, the ear nearly detached, large vessels exposed, coated by small granulations, and sealed up against the passage of blood—yet, I say, with this amount of ulceration and death of parts around, the neighboring glands did not participate in or suffer contamination.

In the cases Nos. 1, 2, and 3, the germ of disease lay, as it were, innocuous; its malignant tendency did not manifest itself until a very advanced period of life, at the respective ages of 52, 70, and 65: while in the cases Nos. 4, 5, and 6, it was ushered into existence at a much earlier age, 19, 28 and 24; while in Mr. Wardrop's case, the subject, a little girl, was only 12 years old.

It is remarkable, too, that once the ulceration was fairly established in the primary tumor, true encephaloid disease rapidly sprung up either in its site or in its immediate locality, with the exception of case No. 4, successfully extirpated. Again, in every instance which I have recorded, all the changes were brought about more speedily, and death followed more quickly, in proportion to the youth of the patient.

The inferences deducible from the results of these several cases, relative to treatment, point to the practical precept of early extirpation; we have evidence of its beneficial results in case No. 4, though ulceration, with its characteristic attendant symptoms, had just manifested themselves; the part was excised, the wound healed, and there has been no return of the disease, though a period of over three years has now elapsed.

In cases Nos. 2, 3, 5, and 6, the operation, I conceive, was had recourse to after the lymphatics and capillaries were charged with the product of the cancerous alteration; and though in some instances the wounds readily healed, yet in a short time the secondary results, the effects of the absorption, manifested themselves in the form of encephaloid disease. So firmly convinced am I of the line of treatment to be adopted in these cases, that I would advise all warts, when situated on the face or elsewhere, to be removed by the knife as early as possible, no matter how youthful the patient may be, as they all have a tendency in advancing years to degenerate in the manner which I have endeavored to represent and elucidate.

Dr. Beatty thought that the cases brought under the noticeof the Society that evening by Mr. Butcher, were both instructive and interesting in the highest degree, and said he fully concurred in his concluding observation, which shoud be borne in mind by every person who was likely to encounter those remarkable affections—namely, that a suspicious looking wart, especially on the face, should no be tampered with by attempting to effect a cure, but should be extirpated with the least possible delay. The remarks of Mr. Butcher had brought to his recollection the case of an acquaintance of his own, a very handsome young lady, who had upon her right cheek a wart of a very suspicious nature. Meeting her in the street one day, he asked her about it, and she informed him that it was a thing of no consequence whatever, and that her apothecary was daily in the habit of applying caustic to the wart with a view of curing it. He advised her to go to some eminent surgeon and have it removed; she took his advice; the wart was extirpated, and from that day to the present there was no return of the disease. But if she had permitted more time to pass without the removal of the excrescence, she would in all probability be now in the condition of one of the unfortunate patients described by Mr. Butcher in his interesting communication.

Dr. Jacob believed the cases adduced by Mr. Butcher to be well worthy the consideration of the profession; and trusted that they would have the effect of calling attention to the subject, as it might perhaps lead to more distinct arrangement of these affections, than had been hitherto made. Last year he (Dr. Jacob) exhibited to the Society a case in which he had extirpated a tumor of a malignant nature from the orbit, and as such a case must be watched with great care, of course he kept the patient within view. It was one of carcinomatous tumor of the orbit, with encephaloid growth engrafted upon it. The poor man had since come back to him with a return of the disease, a great development of encephaloid structure having taken place in the orbit and on the side of the head. The instructive question in those cases was, whether they ought or ought not to extirpate the disease. Eighteen months had gone by since the tumor was removed in the case to which he referred; and therefore he thought that the argument for the operation amounted to this, that the man's life was prolonged, though not saved by it.—*Dublin Medical Press.*

[The foregoing eminently practical paper, well deserves the attentive perusal of the Surgeon. Dr. Butcher has opened up a field for inquiry not hitherto cultivated, and we doubt not, our readers will feel as grateful for his investigations, as we have much pleasure in acknowledging that we are.]—*Eds. Can. Med. Journal.*

